# Case report: Bevacizumab-induced cerebrovascular events: a case series report and literature review

**DOI:** 10.3389/fonc.2025.1395129

**Published:** 2025-02-10

**Authors:** Ke Chen, Boyang Jiang, Huiping Yan, Liu Yang, Zheling Chen

**Affiliations:** ^1^ Department of Gastrointestinal Surgery, The Second Affiliated Hospital, Zhejiang University School of Medicine, Hangzhou, China; ^2^ Cancer Center, Department of Medical Oncology, Zhejiang Provincial People’s Hospital (Affiliated People’s Hospital), Hangzhou Medical College, Hangzhou, Zhejiang, China; ^3^ The Clinical Medical College, Hangzhou Normal University, Hangzhou, Zhejiang, China; ^4^ Department of Medical Oncology, Zhejiang Provincial People’s Hospital, Affiliated People’s Hospital, Hangzhou Medical College, Hangzhou, Zhejiang, China

**Keywords:** bevacizumab, adverts effects, VEGF, cerebrovascular accident, cancer, case report

## Abstract

In clinical use, bevacizumab has improved the overall survival (OS) and progression-free survival (PFS) of malignant solid tumors, and the safe use of bevacizumab has gradually become the mainstream direction of clinical, nursing and pharmaceutical research. Bevacizumab is a humanized anti-VEGF drug. By binding to VEGF, it loses the opportunity to activate VEGFR and then plays an anti-angiogenic role. In addition, more and more studies have emphasized the efficacy and safety of bevacizumab in the treatment of brain metastases from solid tumors. Bevacizumab has its advantages in terms of crossing the blood-brain barrier, increasing radiosensitivity, and reducing radiation-induced brain edema. However, VEGF can maintain the normal function of vascular endothelial cells. Blocking the VEGF pathway can lead to endothelial dysfunction, and the anti-VEGF mechanism of bevacizumab will inevitably lead to a series of thrombotic events and bleeding events. This study reported six cases of cerebrovascular accidents, including ischemic stroke and cerebral hemorrhage, after bevacizumab use. At the same time, this study reviewed the related studies of cardiovascular and cerebrovascular accidents caused by bevacizumab, and analyzed the management of such bevacizumab-related adverse events. We put forward our own views on the early identification and long-term management of adverse events, as well as the subsequent reuse of bevacizumab, hoping to provide more basis for the management of clinical bevacizumab application.

## Introduction

**Figure 1:**
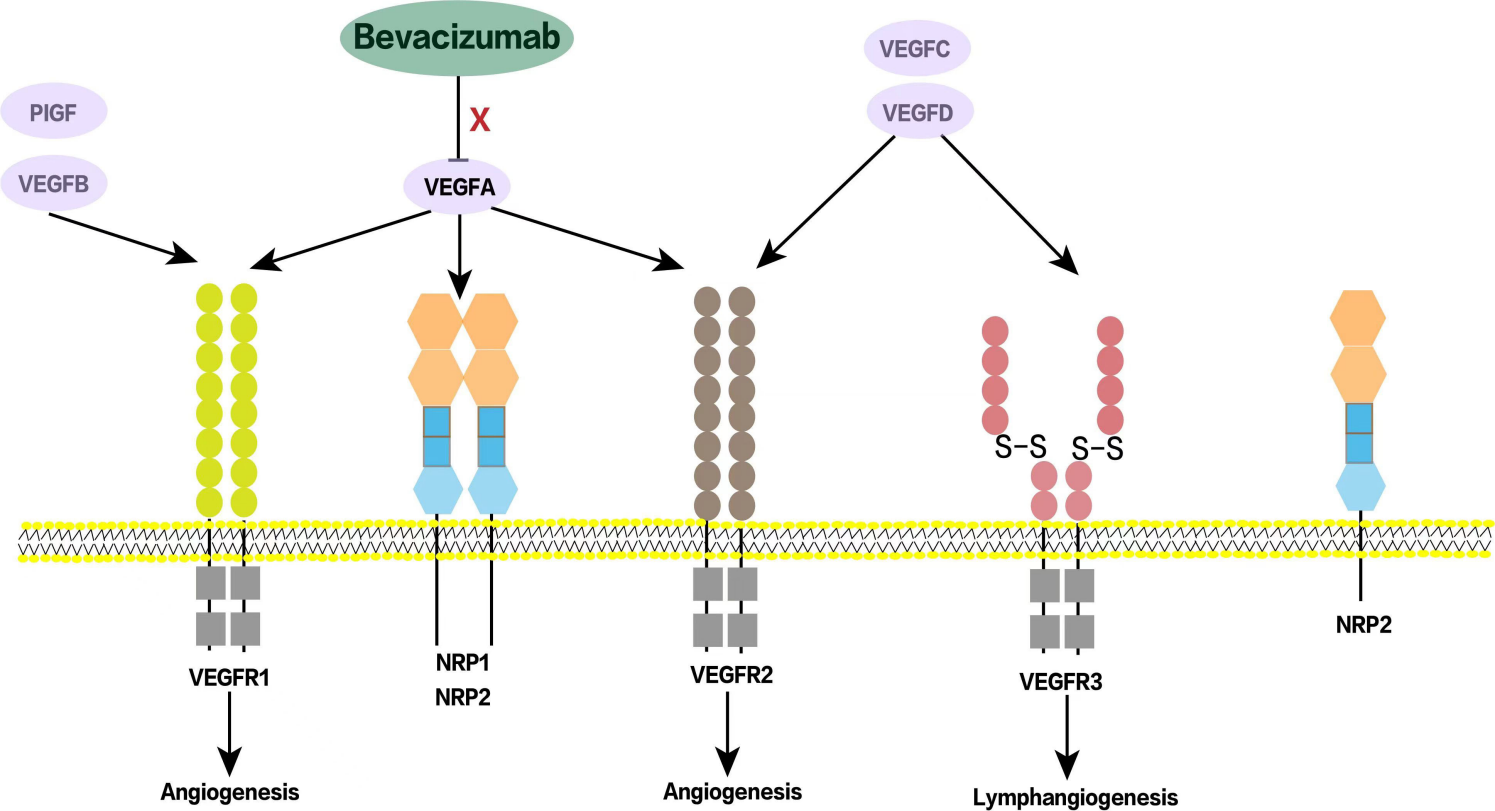
Schematic diagram of bevacizumab acting on VEGF receptors. VEGF family includes VEGF-A, VEGF-B, VEGF-C, VEGF-D and other related factors. Bevacizumab binds to VEGF-A, prevents its interaction with VEGF receptors, plays an anti-angiogenesis role, and then inhibits tumor growth. NRP1/NRP2 are co-receptors of VEGF and also play an important role in tumor angiogenesis. PIGF, Placental growth factor; NRP, Neuropilin.

Bevacizumab is A humanized IgG1 monoclonal antibody that specifically binds to human vascular endothelial growth factor A (VEGFA). By neutralizing VEGF and preventing the activation of VEGF tyrosine kinase receptors VEGFR1 and VEGFR2 on endothelial cells, it attenuates VEGFA-dependent tumor blood vessel formation, promotes tumor vascular normalization and promotes tumor cell apoptosis (1) (**Figure 1**). With the continuous expansion of indications for bevacizumab, more and more patients with solid tumors such as lung cancer, colorectal cancer and cervical cancer have been treated with bevacizumab and their survival has been significantly prolonged (2). Clinical studies have confirmed that on the basis of traditional chemotherapy regimens for a variety of solid tumors, adding bevacizumab not only prolongs progression-free survival (PFS), but also prolongs overall survival (OS), and its treatment has also been promoted from first-line to second-line or even third-line or maintenance therapy, which has become an important weapon in the tumor treatment “toolbox” for the clinicians (3, 4).

Bevacizumab has a half-life of approximately 20 days (2). This provides a time window for the combination of bevacizumab with chemoradiotherapy, but also lays the groundwork for adverse reactions. With the extension of patient survival, clinicians should also pay attention to and manage the adverse reactions of bevacizumab. The four representative adverse reactions of bevacizumab include hypertension, bleeding, proteinuria, and thromboembolism (5).Bevacizumab is an inhibitor of VEGF, which has the function of maintaining normal vascular endothelial cells, so blocking the VEGF pathway can lead to endothelial dysfunction (6).Vascular endothelial cell apoptosis or inhibition of endothelial cell regeneration can destroy the integrity of endothelial cells. The exposure of procoagulant phospholipids and the aggregation of various cytokines can promote thrombosis. In addition, bevacizumab-induced reductions in nitric oxide and prostacyclin levels also lead to platelet aggregation and thrombosis (7). In current bevacizuab-related clinical trials, the overall incidence of arterial thromboembolism in the bevacizumab group has been reported from 0.7% to 8.7%, venous embolism events have been reported from 0.5% to 10.1%, and cerebral hemorrhage events have been reported from 0.0% to 3.8% (**Supplementary Table S1**) (8–14).The clinical trial AVF2192 g included patients with metastatic colorectal cancer who were ineligible for irinotecan. Arterial thromboembolism was observed in 11% (11/100) of patients in the bevacizumab group and in 5.8% (6/104) of patients in the chemotherapy control group in this trial (15).

Although the adverse reactions of bevacizumab are well known in clinical practice, there is a lack of specific analysis of cerebrovascular events caused by bevacizumab in the real world. Cerebrovascular events reported in the literature refer to transient ischemic attacks, cerebral hemorrhage, and ischemic stroke that occur during treatment with bevacizumab. Theoretically, it seems that once such adverse events occur, the drug should be stopped permanently. However, it is difficult to balance the risk of disease progression associated with discontinuation with the risk of thrombosis/bleeding associated with continued treatment. At present, there are few research data to guide the clinical use of anticoagulant drugs during the use of bevacizumab, and the above problems need to be further explored in clinical practice. “Here, we report six cases of cerebrovascular events after the administration of bevacizumab at doses ranging from 5 to 7.5 mg/kg.

## Patients and methods

This study was a retrospective study, and the enrollment criteria were: (1) clear diagnosis of malignant tumor; (3) Cerebrovascular accidents occurred during the course of bevacizumab treatment, including transient ischemic attack, cerebral hemorrhage and ischemic stroke, which were specifically diagnosed with brain CT or brain MRI imaging support; (4) Complete case data.

Exclusion criteria were as follows: (1) The interval between bevacizumab use and cerebrovascular accident was more than 6 half-lives of bevacizumab or before bevacizumab use was judged to be unrelated to bevacizumab use. (2) Incomplete case data; (3)The patients had basic cerebrovascular malformations or previous cerebrovascular accidents;(4)Underlying uncontrolled severe hypertension.

**Figure 2:**
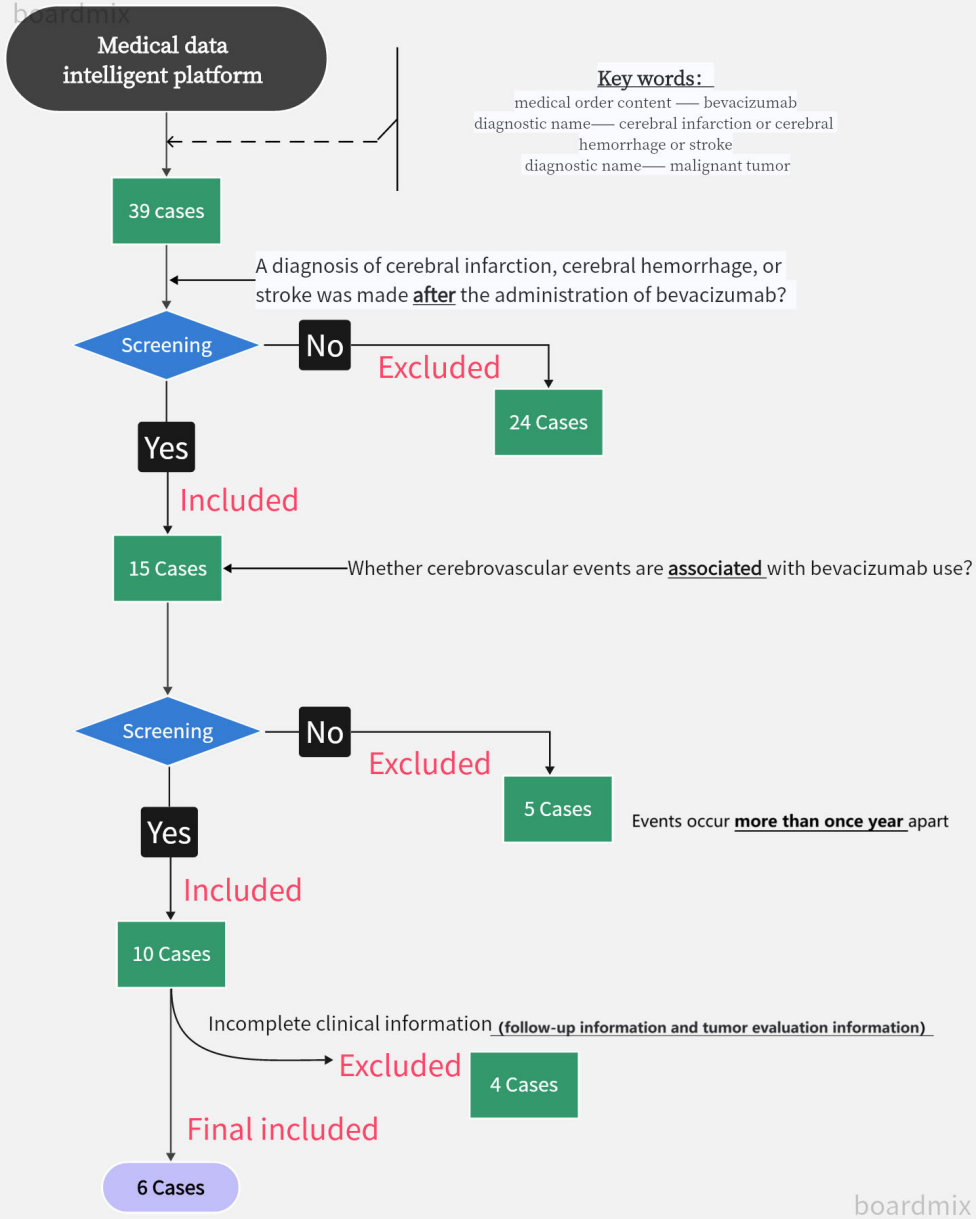
Algorithm for patient screening.

We reviewed all patients with malignant tumors treated at Zhejiang Provincial People’s Hospital from January 2018 to December 2023. From January 2018 to December 2023, there were 5,584 patients with malignant tumors in the unit, and 2,157 patients received bevacizumab. The medical data intelligent platform was used for case information retrieval in our unit. The search keywords included “medical order content - bevacizumab”, “diagnostic name - cerebral infarction or cerebral hemorrhage or stroke”, “diagnostic name - malignant tumor”. A total of 39 cases were retrieved, of which 24 cases with cerebral infarction, cerebral hemorrhage or stroke diagnosis before the use of bevacizumab were excluded. In humans, the final half-life of bevacizumab is 17-21 days. Five patients were treated with bevacizumab only once during the treatment, and their cerebral infarction, cerebral hemorrhage or stroke occurred at an interval of more than 1 year after the treatment (far exceeding 6 half-lives of bevacizumab), which was judged to be irrelevant to bevacizumab and excluded. Four patients with incomplete clinical information were excluded (follow-up information and tumor evaluation information), and 6 patients were finally included for analysis. The specific retrieval process is shown in **Figure 2**.

The median overall survival (OS) of all patients was 54 months (12-66 months), and the median bevacizumab-related PFS was 5 months (3-28 months). The basic information of all patients, history of underlying diseases, dose of bevacizumab, concomitant treatments, and treatment for cerebrovascular accidents are summarized in **Table 1**. The six patients, whose tumors included lung, rectal, and endometrial cancers, ranged in age from 46 to 69 years and had no history of cerebrovascular disease. Two had a history of hypertension but were well controlled on medication. The dose of bevacizumab was 5.0-7.5mg/kg. One patient had a cerebrovascular accident and stopped taking bevacizumab (P02) after one dose, and the duration of bevacizumab used in the remaining patients ranged from 3 to 28 months. P01-P05 had cerebral infarction and were treated with anticoagulation. The patient P06 suffered from cerebral hemorrhage, and was treated with blood pressure control and supportive treatment. Representative images of ischemic stroke/cerebral hemorrhage in 6 patients are shown in **Supplementary Figure S1**.

**Table 1:** Basic clinical information of the patients.

Case No.	Sex	Age	Tumor type	Previous cardio-cerebrovascular disease	History of hypertension	Brain metastasis	Gamma knife radio-surgery	Bev. Dose and cycle	Combination therapy with Bev.	Duration of bev. use^2^	Major cardiovascular envents(MACE)^1^	Treatment of MACE	Other AEs
1	Female	69	NSCLC	None	None	Yes	Yes	7.5mg/kg, q3w	Pemetrexed,Oxitinib,Fumetinib	13 months	Lacunar infraction	Rivaroxaban, aspirin	Proteinuria (I°); heypertesion (II°)
2	Male	61	SCLC	None	None	Yes	Yes	5mg/kg, q3w	None	1 day	Cerebral infraction	Aspirin, atorvastatin	None
3	Female	63	NSCLC	None	None	Yes	Yes	7.5mg/kg	Pemetrexed,gefitinb	28 months	Cerebral infraction	atorvastatin	Proteinuria (I°)
4	Male	65	Rectal cancer	None	None	None	None	5mg/kg, q2w	Folfiri, Tas-102	9 months	Cerebral infraction	atorvastatin	Proteinuria (I°)
5	Female	46	NSCLC	None	Yes	None	None	5mg/kg, q3w	Pemetrexed	5 months	Cerebral infraction	Aspirin	None
6	Female	57	Endometrial cancer	None	Yes	None	None	5mg/kg, q3w	Paclitaxel, carboplatin	3 months	Cerebral hemorrage	Antihypertensive and supportive treatment	heypertesion (III°)

**Figure 3:**
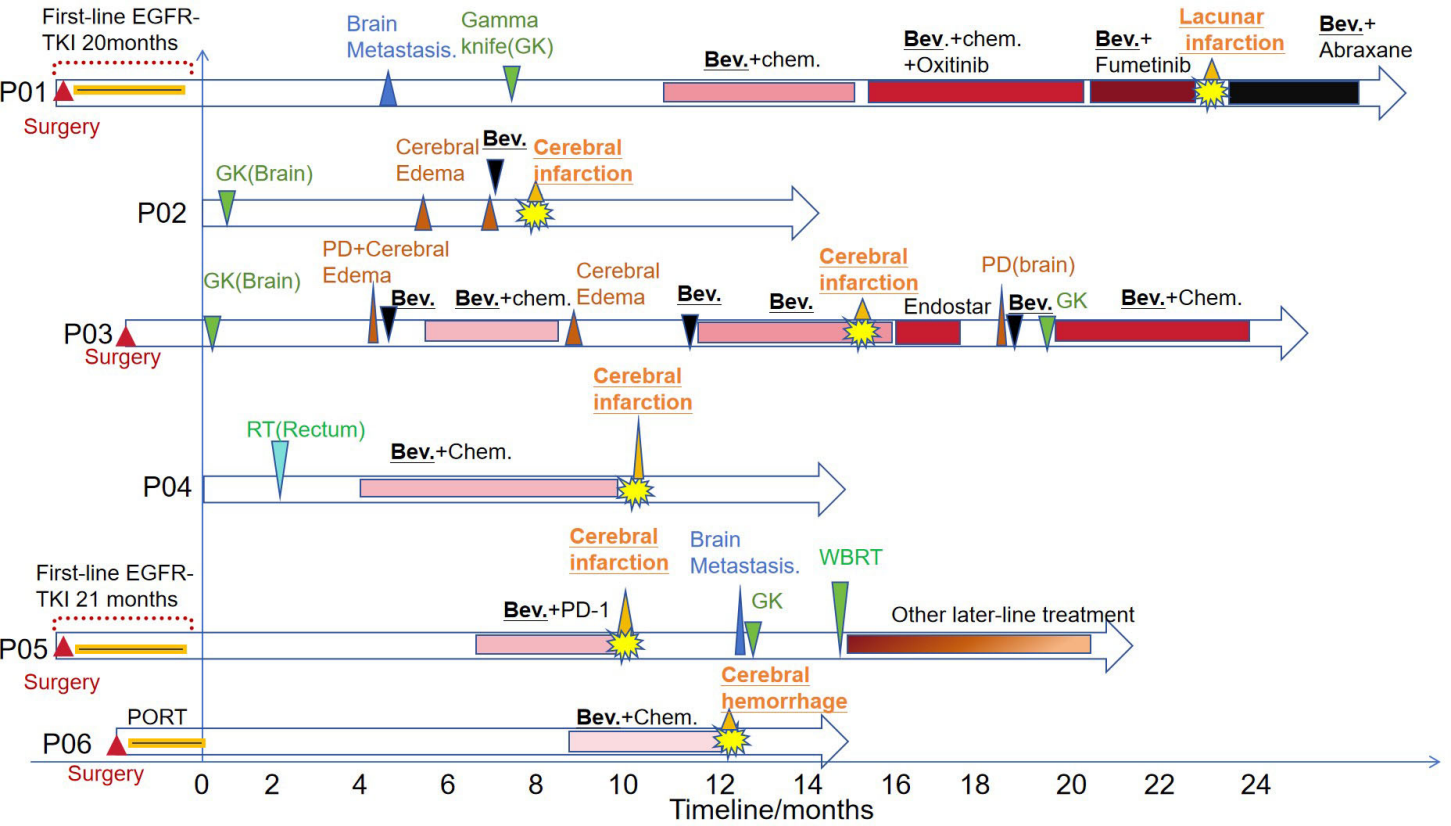
The treatment timeline of the six patients. PORT, Postoperative Radiotherapy; TKI, tyrosine kinase inhibitor; RT, radiotherapy; Chem., Chemotherapy; GK, Gamma knife; Bev., Bevacizumab; PD, progressive disease; WBRT, whole brain radiation therapy.

These 6 patients included 4 cases of lung cancer (P01, P02,P03 and P05), 1 case of rectal cancer (P04) and 1 case of cervical cancer (P06). Four lung cancer patients had brain metastases and all of them were treated with gamma knife. Patients P02 and P03 had brain edema after gamma knife treatment. Three patients with lung cancer were treated with bevacizumab after gamma knife treatment (P01,P02 and P03) and subsequently had cerebrovascular events at various time points (1 day to 28 months). P01 and P03 continued bevacizumab in subsequent treatment after symptom recovery, while P02 left severe hemiplegia. Bevacizumab was permanently discontinued. Patient P04, a rectal cancer patient, had a cerebral infarction during the crossover of bevacizumab, and bevacizumab was permanently discontinued in the subsequent treatment. Patient P06 was a cervical cancer patient who developed cerebral hemorrhage during the use of bevacizumab and subsequently discontinued bevacizumab. The treatment timeline for patients is shown in **Figure 3**.

## Discussion

Angiogenesis is a fundamental activity in the process of tumor growth and metastasis (16).Therefore, in the field of cancer research, there is great interest in studying the molecular mechanisms of tumor angiogenesis. The vascular endothelial growth factor (VEGF) pathway is a key regulator of this process. The role of VEGF in promoting tumor angiogenesis, which promotes tumor generation, development, and migration (17). Therefore, it is necessary to design and develop inhibitors targeting this pathway. Bevacizumab binds VEGF and prevents its binding to receptors on the surface of vascular endothelial cells, thereby inhibiting angiogenesis in malignant tumors (18). In contrast to chemotherapeutic agents, bevacizumab does not produce typical cytotoxic effects and does not increase chemotherapy-related toxicity when combined with chemotherapy. However, the use of bevacizumab as an antiangiogenic therapy may be associated with vascular-related adverse events, including embolization and bleeding.Bevacizumab is thought to be associated with an increased risk of arterial thromboembolic events (19), and its susceptibility to venous thrombosis in the vein remains controversial.The incidence of VTE in patients treated with bevacizumab reported in the literature ranged from 3% to 19.4% (20, 21).A meta-analysis showed that the use of bevacizumab significantly increased the risk of venous thromboembolism in cancer patients (RR=1.33) (22). Bleeds associated with bevacizumab were mostly minor epistaxis and other self-limiting mucosal bleeds. But there have also been reports of serious (≧̸ grade 3) bleeding events (23).In addition to pulmonary hemorrhage, it also included severe gastrointestinal (GI) hemorrhage, urogenital tract hemorrhage, and central nervous system (CNS) hemorrhage (24, 25).Vascular events in the CNS include intracranial hemorrhage and ischemic stroke (26).

Mechanistically, bevacizumab can damage the integrity of vascular endothelial cells, inhibit the expression of prostaglandin and nitric oxide, and promote platelet aggregation, thereby increasing the risk of ischemic cerebrovascular events. In addition, bevacizumab can also inhibit the proliferation and migration of endothelial cells, which may lead to damage to vascular integrity, resulting in endothelial dysfunction and bleeding (27).

In our report, three patients with lung cancer were treated with bevacizumab after gamma knife treatment (P01,P02, and P03), and cerebral edema developed in two patients. Through our conversations with the clinicians, we learned that bevacizumab was administered largely for the treatment of radiation-induced cerebral edema, and then the patient unfortunately had a cerebrovascular accident in the subsequent course of the disease. Some studies have suggested that bevacizumab can normalize blood vessels in brain metastases, reduce vascular endothelial permeability, and improve peritumoral edema, thereby alleviating symptoms in patients with brain metastases (28–30).In 2009, the PASSPORT study established the safety of bevacizumab in NSCLC patients who had been treated with either radiation or surgery for brain metastases (31).This study showed that bevacizumab combined with chemotherapy or erlotinib for NSCLC patients with brain metastases was safe and had a low incidence of cerebral hemorrhage. Retrospective analyses from both U.S. and European databases have shown that the incidence of symptomatic intracranial hemorrhage in patients with NSCLC and brain metastases is comparable, at less than 5%, with or without bevacizumab. Therefore, on March 25, 2009, the European Union removed brain metastases as a contraindication for bevacizumab treatment. According to the 2022 Chinese expert consensus on anti-angiogenic drugs for advanced non-small cell lung cancer, bevacizumab can significantly relieve the symptoms caused by peritumoral edema and reduce the degree of edema on imaging in advanced NSCLC patients with symptomatic brain metastases such as brain edema or brain necrosis (Grade II recommendation, Class 2A evidence). However, the specific dosage has not been clearly prescribed, and the commonly used dose is 5-10 mg/kg, once every 2-3 weeks, 2-6 courses of treatment (32, 33). Although a number of clinical studies and expert consensus have recommended the application of bevacizumab in patients with brain metastases, and bevacizumab has the advantage of crossing the blood-brain barrier, the risk of vascular-related adverse events still cannot be ignored. Although bevacizumab has largely prolonged survival in such patients, cerebrovascular events can occur as soon as 1 day after treatment, and the most severe patients are left with permanent impairment of physical activity. Early identification, prevention, and education of vascular events remain important in the clinical use of bevacizumab.

There is some controversy about whether bevacizumab can be continued after the occurrence of vascular adverse events. It is currently believed that in the event of a serious thrombotic event, bevacizumab should be discontinued and anticoagulant therapy should be given. Once an arterial thromboembolic event (ATE) occurs, bevacizumab therapy should be discontinued in the acute phase. Patients with a recent ATE should not be treated with bevacizumab for at least 6 months after the onset of ATE, and the patient should be determined to be stable or asymptomatic before initiating bevacizumab therapy (27). Ischemic stroke can be considered a serious arterial thromboembolic event, and the two patients in this case who continued bevacizumab after recovery from stroke had good disease control and did not worsen ATE during long-term follow-up. In the real world, anti-angiogenic therapy is the cornerstone of many solid tumors, including lung cancer and intestinal cancer. Continuous anti-angiogenic therapy effectively prolongs the survival of patients throughout the treatment. In our center, The number of patients using beva in this center is about 2350 a year. Therefore, through close follow-up and observation, evaluating the risk of patients, weighing the pros and cons of re-using bevacizumab, and effective management of side effects, we can better play the efficacy of bevacizumab and bring more valuable treatment hope for cancer patients.

## Conclusion

In real-world clinical practice, bevacizumab is widely used in patients with solid tumors, especially brain metastases. However, vascular accidents caused by bevacizumab, especially cerebrovascular accidents, deserve clinical attention, and serious cases can leave irreversible sequelae. As a macromolecular drug, the efficacy of bevacizumab in the treatment of intracranial lesions has been controversial. Whether the risk of cerebrovascular accident is higher in patients with intracranial lesions is worthy of further discussion. Our study included cases of cerebrovascular accident following bevacizumab treatment, including patients with brain metastases, and fully evaluated the association between bevacizumab use and cerebrovascular accident. This study further reviewed previous studies to discuss the occurrence and management of bevacizumab related cerebrovascular accidents, so as to provide a basis for clinical practice.

## Data Availability

The original contributions presented in the study are included in the article/**Supplementary Material**. Further inquiries can be directed to the corresponding author.

## Supplementary material

Supplementary Figure 1 The cranial imaging of cerebralvascular accidents. **(A)** P01, acute lacunar cerebral infarction in the left hemioval region. **(B)** P02, acute cerebral infarction was considered in the right paraventricular basal ganglia. **(C)** P03, multiple abnormal signal foci around the left ventricle and basal ganglia were considered acute infarction. **(D)** P04, large cerebral infarction was observed in the left temporal lobe, frontal lobe, Broca area, and lateral ventricle. **(E)** P05, cerebral infarction in the left parietal lobe. **(F)** P06, cerebral hemorrhage in the left basal ganglia.
