# Supplementary figures and images for: Case report: Bevacizumab-induced cerebrovascular events: a case series report and literature review
Source: Front Oncol. 2025 Feb 10;15:1395129. doi: 10.3389/fonc.2025.1395129 (PMC11847825; doi:10.3389/fonc.2025.1395129)

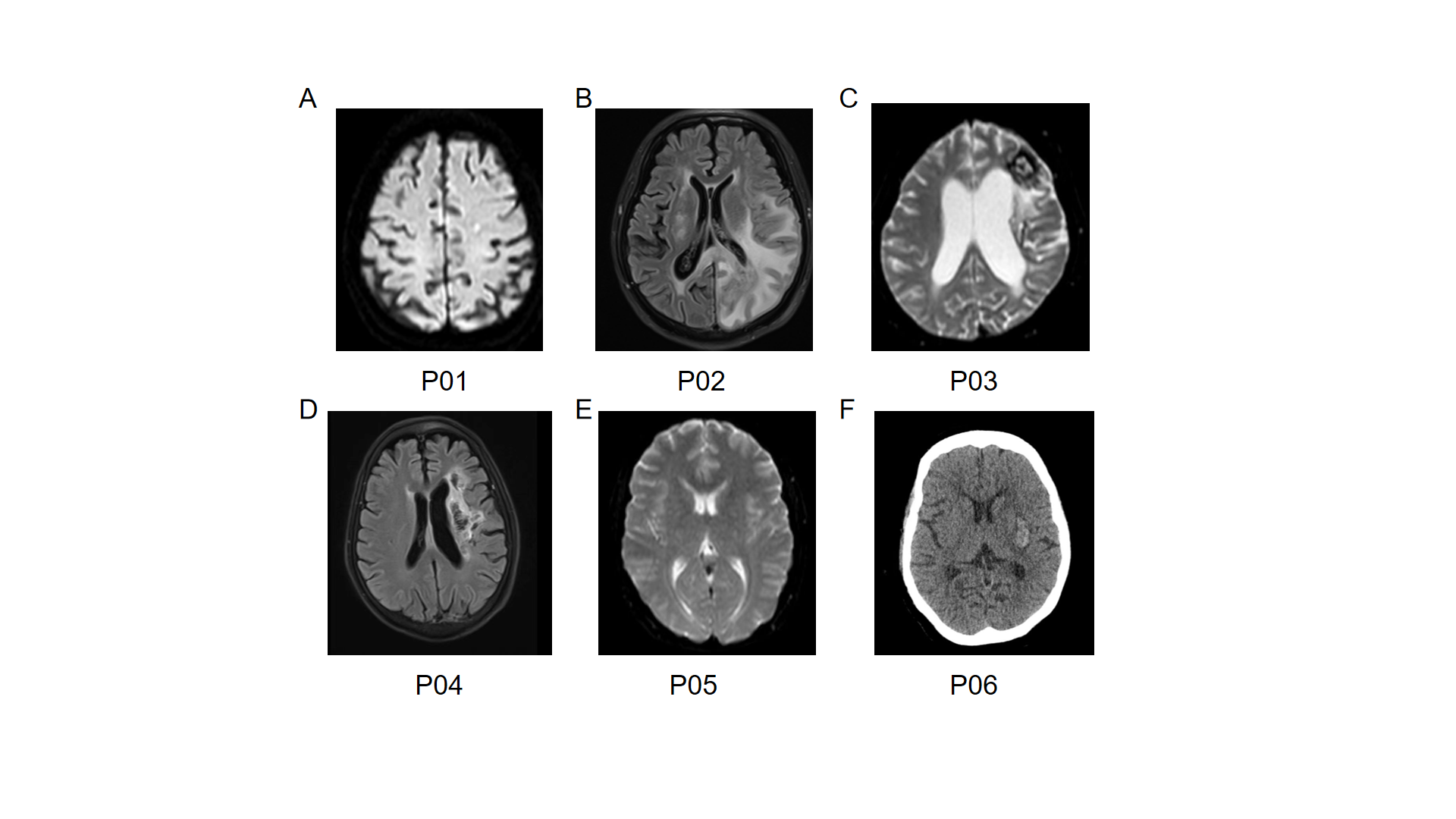

Supplement: Supplementary Figure 1 — The cranial imaging of cerebralvascular accidents. (A) P01, acute lacunar cerebral infarction in the left hemioval region. (B) P02, acute cerebral infarction was considered in the right paraventricular basal ganglia. (C) P03, multiple abnormal signal foci around the left ventricle and basal ganglia were considered acute infarction. (D) P04, large cerebral infarction was observed in the left temporal lobe, frontal lobe, Broca area, and lateral ventricle. (E) P05, cerebral infarction in the left parietal lobe. (F) P06, cerebral hemorrhage in the left basal ganglia. [file Image1.tiff]
