# Supplementary material for: Case report: Bevacizumab-induced cerebrovascular events: a case series report and literature review
Source: Front Oncol. 2025 Feb 10;15:1395129. doi: 10.3389/fonc.2025.1395129 (PMC11847825; doi:10.3389/fonc.2025.1395129)
Supplement: Supplementary file 2 [file Table1.docx]

**Supplementary Table 1 Summary of some adverse reactions in clinical trials related to bevacizumab**

| **Studys** | **Olivier L Chinot et al.[8]** | **Henry S. Friedman et al.[9]** | | **Kathy Miller et al.[10]** | **WJOG4407G[11]** | | **Krishnansu S Tewari et al.[12]** | **Krishnansu S Tewari et al.[13]** | **Timothy J et al.[14]** |
| --- | --- | --- | --- | --- | --- | --- | --- | --- | --- |
| **Side Effects**  **Groups** | Bevacizumab plusRadiotherapy andTemozolomide | Bevacizumab | Bevacizumab plus Cpt-11 | Paclitaxel plus Bevacizumab | FOLFIRI plus bevacizumab | mFOLFOX6 plus bevacizumab | Chemotherapy plusBevacizumab | Bevacizumab arm | Bevacizumab |
| Hemorrhage | / | / | / | 0.5%* | 11.8% | 6.6% | 5.0% | 2.4% | 39.0% |
| Cerebral hemorrhage | 3.3% | 2.4% | 3.8% | / | / | / | 0.0% | 0.3% | 0.2% |
| Cerebrovascular ischemia | / | / | / | 1.9%* | / | / | / |  | / |
| Thromboembolicdisease | / | / | / | 2.1%* | / | / | / | 6.9% | / |
| Arterial thromboembolic events§ | 5.9% | 4.8% | 6.3% | / | 8.7% | 4.5% | / | 0.7% | / |
| Venous thromboembolic events | 8.2% | 3.6% | 10.1% | / | 2.0% | 0.5% | / | / | / |

*Grade 3 and Grade 4
